# The complete mitochondrial genome of *Flustra foliacea *(Ectoprocta, Cheilostomata) - compositional bias affects phylogenetic analyses of lophotrochozoan relationships

**DOI:** 10.1186/1471-2164-12-572

**Published:** 2011-11-23

**Authors:** Maximilian P Nesnidal, Martin Helmkampf, Iris Bruchhaus, Bernhard Hausdorf

**Affiliations:** 1 Zoological Museum of the University of Hamburg, Martin-Luther-King-Platz 3, 20146 Hamburg, Germany; 2 Bernhard Nocht Institute for Tropical Medicine, Bernhard-Nocht-Strasse 74, 20359 Hamburg, Germany; 3 School of Life Sciences, Arizona State University, 427 East Tyler Mall, Tempe, AZ 85287, USA

## Abstract

**Background:**

The phylogenetic relationships of the lophophorate lineages, ectoprocts, brachiopods and phoronids, within Lophotrochozoa are still controversial. We sequenced an additional mitochondrial genome of the most species-rich lophophorate lineage, the ectoprocts. Although it is known that there are large differences in the nucleotide composition of mitochondrial sequences of different lineages as well as in the amino acid composition of the encoded proteins, this bias is often not considered in phylogenetic analyses. We applied several approaches for reducing compositional bias and saturation in the phylogenetic analyses of the mitochondrial sequences.

**Results:**

The complete mitochondrial genome (16,089 bp) of *Flustra foliacea *(Ectoprocta, Gymnolaemata, Cheilostomata) was sequenced. All protein-encoding, rRNA and tRNA genes are transcribed from the same strand. *Flustra *shares long intergenic sequences with the cheilostomate ectoproct *Bugula*, which might be a synapomorphy of these taxa. Further synapomorphies might be the loss of the DHU arm of the tRNA L(UUR), the loss of the DHU arm of the tRNA S(UCN) and the unique anticodon sequence GAG of the tRNA L(CUN). The gene order of the mitochondrial genome of *Flustra *differs strongly from that of the other known ectoprocts. Phylogenetic analyses of mitochondrial nucleotide and amino acid data sets show that the lophophorate lineages are more closely related to trochozoan phyla than to deuterostomes or ecdysozoans confirming the Lophotrochozoa hypothesis. Furthermore, they support the monophyly of Cheilostomata and Ectoprocta. However, the relationships of the lophophorate lineages within Lophotrochozoa differ strongly depending on the data set and the used method. Different approaches for reducing heterogeneity in nucleotide and amino acid data sets and saturation did not result in a more robust resolution of lophotrochozoan relationships.

**Conclusion:**

The contradictory and usually weakly supported phylogenetic reconstructions of the relationships among lophotrochozoan phyla based on mitochondrial sequences indicate that these alone do not contain enough information for a robust resolution of the relations of the lophotrochozoan phyla. The mitochondrial gene order is also not useful for inferring their phylogenetic relationships, because it is highly variable in ectoprocts, brachiopods and some other lophotrochozoan phyla. However, our study revealed several rare genomic changes like the evolution of long intergenic sequences and changes in the structure of tRNAs, which may be helpful for reconstructing ectoproct phylogeny.

## Background

Molecular systematics has dramatically changed the ideas about the phylogenetic relationships of the lophophorate lineages, ectoproct bryozoans, brachiopods and phoronids. Once considered the sister or paraphyletic stem-group of Deuterostomia based on embryological and morphological characters [1-5], molecular analyses almost unequivocally place them in Lophotrochozoa, a group established to accommodate the lophophorate lineages along with trochozoans, Annelida, Mollusca and relatives [6]. These analyses rely on a spectrum of molecular data ranging from rDNA sequences [6-16], mitochondrial protein sequences [17-19], single nuclear protein-encoding genes [20,21], *Hox *genes [22,23], multiple nuclear protein-encoding sequences [24,25] and phylogenomic approaches [26-34].

However, the relationships of the lophophorate lineages within Lophotrochozoa are still controversial, because analyses of different data sets resulted in conflicting topologies. Whereas analyses of rDNA data sets [7,8,14-16,35,36] and phylogenomic data sets [27,29,34] strongly support Brachiozoa, a clade including Brachiopoda and Phoronida, phylogenetic analyses of mitochondrial sequences most often indicated sister group relationships between Brachiopoda and Ectoprocta [19,37-39] and between Phoronida and Entoprocta [37-39]. Thus, these analyses also contradict the monophyly of Bryozoa (= Polyzoa) including Ectoprocta and Entoprocta (and Cycliophora, of which no mitochondrial genome is available so far) as postulated based on morphological arguments by Nielsen [40,41] and found in some recent analyses of phylogenomic data sets [26,29-34] and of rDNA data sets [14-16], albeit with poor nodal support.

There is evidence that the inference of the relationships of the lophophorate lineages in phylogenomic analyses might be affected by systematic errors resulting from compositional bias [34]. One possibility to check for systematic errors in phylogenetic analyses is the comparison of the results based on independent data sets. Therefore, we analysed a mitochondrial data set in this study and compared the phylogenetic results with those of phylogenomic analyses, in which no or only few mitochondrial data have been considered. We sequenced an additional mitochondrial genome of the most species-rich lophophorate lineage, the ectoprocts. Because there are large differences in the nucleotide composition of mitochondrial sequences of different lineages as well as in the amino acid composition of the encoded proteins [42-48], we applied several approaches for reducing compositional bias in the phylogenetic analyses. We reduced the compositional heterogeneity by excluding third codon positions from the nucleotide data set, by excluding taxa with strongly deviating amino acid composition and by recoding amino acids in bins. As an alternative to reducing compositional heterogeneity in the data, we applied phylogenetic inference methods with nonstationary models of evolution. Finally, we tried to mitigate saturation and long-branch-attraction problems by excluding fast evolving sites.

## Results and Discussion

### Organization of the mitochondrial genome of the ectoproct Flustra foliacea

**Figure 1:**
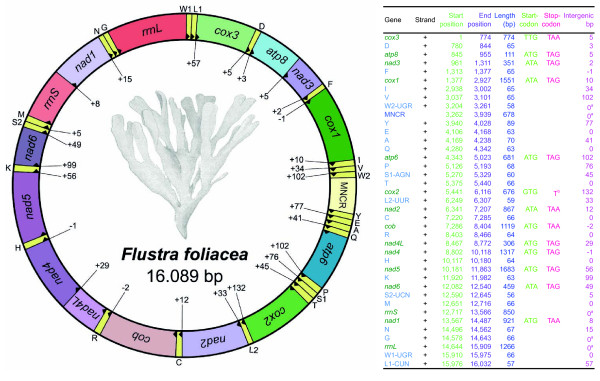
**Structure of the mitochondrial genome of *Flustra foliacea*(GenBank accession number JQ061319).** The arrows indicate the direction of transcription. Numbers indicate noncoding nucleotides between genes (negative values refer to gene overlaps). The tRNA genes are named using single-letter amino acid abbreviations. Those coding for leucine, serine and tryptophan are named L1 for the tRNA^Leu(CUN) ^(anticodon UAG) gene, L2 for the tRNA^Leu(UUR) ^(anticodon UAA) gene, S1 for the tRNA^Ser(AGN) ^(anticodon UCU) gene, S2 for the tRNA^Ser(UCN) ^(anticodon UGA) gene, and W1 for the tRNA^Trp(UGR) ^(anticodon UCA) gene and W2 for the tRNA^Trp(UGR) ^(anticodon UCA) gene. The genomic features are described in the table on the right. ^a^: Start and end positions of rRNA genes and MNCR determined by boundaries of adjacent genes. ^b^: Incomplete termination codon, which is probably extended by post-transcriptional adenylation.

**Figure 2:**
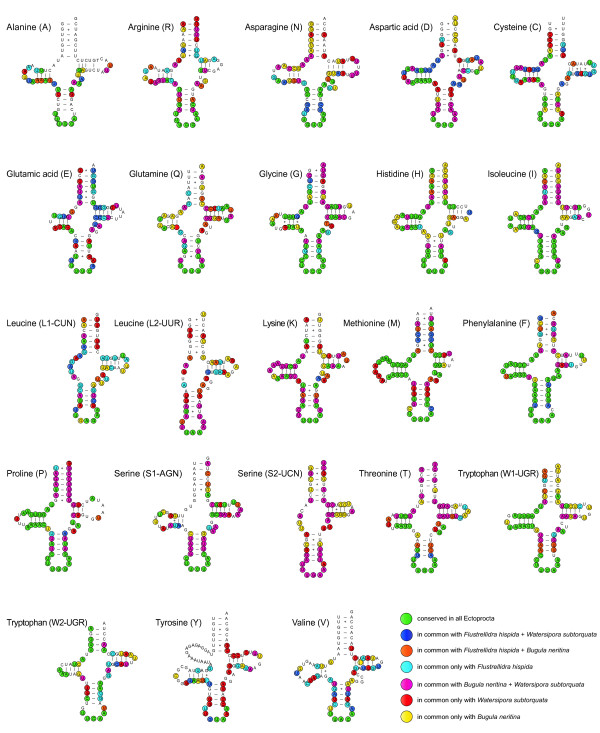
**Putative secondary structures of the 23 tRNAs identified in the mitochondrial genome of *Flustra foliacea***. Bars indicate Watson-Crick base pairings, and crosses between G and U pairs mark canonical base pairings appearing in RNA.

The mitochondrial genome sequence of the ectoproct *Flustra foliacea *(Gymnolaemata, Cheilostomata) is 16,089 bp long and consists of 13 protein-encoding genes (*atp6*, *atp8*, *cox1-3*, *cob*, *nad1-nad6 *and *nad4L*) and two rRNA genes for the small and large subunits (*rrnS *and *rrnL*), as is typical for animal mitochondrial genomes (Figure 1). In addition to the 22 usual tRNA genes (Figure 2), a second putative tRNA gene for tryptophan is found. All protein-encoding, rRNA and tRNA genes are transcribed from the same strand, as is the case with the protein-encoding and rRNA genes of the other cheilostomate ectoprocts with known mitochondrial genomes, *Bugula neritina *[38] and *Watersipora subtorquata *[49]. There is a major non-coding region (678 bp long) with a high A+T content of 65.8%, which might be the origin of replication. However, as in *Bugula*, there are several additional long intergenic sequences (Figure 1) that sum up to 997 bp; 16 of them are longer than 10 bp, the maximum being 132 bp. Such long intergenic sequences are missing in *Watersipora *and the ctenostomate *Flustrellidra *[19]. Thus, they might be synapomorphies of the lineages leading to *Flustra *and *Bugula*. However, no conserved sequence motifs could be identified by blast searches with the noncoding regions of *Flustra *against the noncoding regions of *Bugula*.

### Transfer RNA genes

A second putative tRNA gene for tryptophan as found here in *Flustra foliacea *(Figure 2) has neither been found in the other known mitochondrial genomes of ectoprocts nor in most other animal mitochondrial genomes. There is no similarity between the sequence of this putative tRNA gene and any of the other tRNA genes in the mitochondrial genome of *Flustra*. It is proximate to the major non-coding region. We cannot exclude the possibility that it is functionally part of the control region. Nevertheless, its structure is very similar to a tRNA and it is likely that it is at least derived from a tRNA. The two leucine and one of the serine tRNAs lack a DHU arm. The DHU arm of the tRNA L(UUR) is also missing in the cheilostomate *Bugula*, but not in the cheilostomate *Watersipora *and the ctenostomate *Flustrellidra*, whereas the DHU arm of the tRNA L(CUN) is also missing in *Flustrellidra*, but not in *Bugula *and *Watersipora*. Given the relations of these taxa, the loss of the DHU arm of the tRNA L(UUR) might be a synapomorphy of the lineages leading to *Flustra *and *Bugula*, whereas the loss of the DHU arm of the tRNA L(CUN) occurred most likely independently in *Flustra *and *Flustrellidra*. The DHU arm of the tRNA S(UCN) is also missing in *Bugula*, but not in *Watersipora *and might be another synapomorphy of the lineages leading to *Flustra *and *Bugula*. This tRNA has not been found in *Flustrellidra*.

The inferred anticodons of 21 tRNAs of *Flustra foliacea *(Figure 2) are the same as those in *Bugula neritina*. Only the anticodon of the tyrosine tRNA differs between *Flustra *and *Bugula*. The anticodon of tyrosine tRNA is GUA in *Flustra*, but AUA in *Bugula*. Because the anticodon of the *Watersipora *and *Flustrellidra *tyrosine tRNAs is also GUA, the change to AUA is probably an autapomorphy of the lineage leading to *Bugula*. The anticodon of the tRNA L(CUN) of *Flustra *and *Bugula *is GAG. This has not been found in any other metazoan so far. In *Watersipora *and *Flustrellidra *the anticodon of the tRNA L(CUN) is UAG. Thus, the sequence GAG may represent a unique synapomorphy of the lineages leading to *Bugula *and *Flustra*.

The most conserved region of all tRNAs is the anticodon stem and loop region (Figure 2). All other tRNA regions have a high level of variation within Ectoprocta. Especially the TΨC arm is highly variable. The tRNAs with the highest nucleotide conservation across the four ectoprocts are Gly, His, Ile, Met, Phe, Pro and Trp1. Less conserved tRNAs are Arg, Asn, Ser2, Lys, Thr and Trp2. As expected from the phylogenetic relationships, the tRNA sequences of *Flustra *are most similar to those of the cheilostomate entoprocts *Watersipora *(p-distance based on all concatenated tRNAs equals 0.357) and *Bugula *(p-distance 0.361), whereas those of the cheilostomate *Flustrellidra *are more dissimilar (p-distance 0.461).

### Comparison of mitochondrial gene order

**Figure 3:**
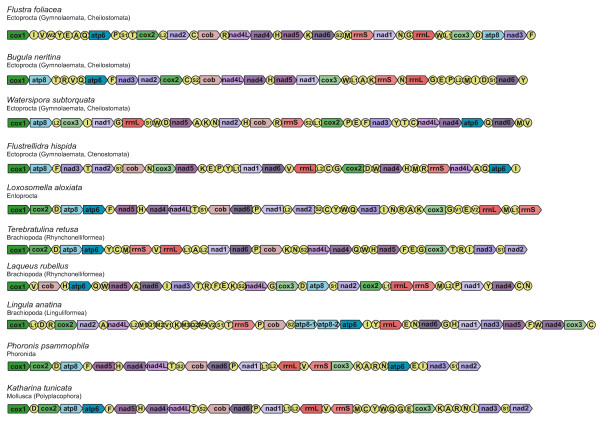
**Comparison of the arrangement of the mitochondrial genes of representatives of ectoprocts, entoprocts, brachiopods, phoronids, and molluscs**. The arrows indicate the direction of transcription. Gene and genome size are not to scale.

The order of the protein-encoding and rRNA genes is highly variable within ectoprocts (Figure 3). The only conserved block in the cheilostomate ectoprocts *Flustra *and *Bugula *including three or more genes is *cob*-*nad4L*-*nad4-nad5*. There is no block of three or more genes with identical order in *Flustra *and the cheilostomate *Watersipora *or the ctenostomate ectoproct *Flustrellidra*. The block *cob*-*nad4L*-*nad4-nad5 *is also present in several other lophotrochozoans, e.g., entoprocts, phoronids, and some molluscs. Thus, it might be a symplesiomorphy within ectoprocts. All breakpoint distances between the three cheilostomate ectoprocts (*Flustra, Bugula *and *Watersipora*) calculated with CREx [50] amount to 12, the breakpoint distances between the three cheilostomate ectoprocts and the ctenostomate ectoproct *Flustrellidra *to 13 and the breakpoint distances between the ectoprocts and other lophophorates and entoproct to 9-15 (Table 1). The breakpoint distances between the three brachiopods are 13-15. Thus, there were so many gene order rearrangements within Ectoprocta and within Brachiopoda that there is almost no chance to reconstruct older rearrangements, which might provide evidence for the relationships of ectoprocts and brachiopods with other lophotrochozoans. In contrast, gene order rearrangements may be useful for inferring phylogeny within ectoprocts and brachiopods. However, a denser taxon sampling is necessary to resolve the sequence of rearrangements that caused the many differences observed within ectoprocts and brachiopods.

**Table 1:** Breakpoint distance matrix between orders of mitochondrial protein coding genes and rDNAs of representatives of ectoprocts, entoprocts, brachiopods, phoronids, and molluscs.

Taxa	*Ff*	*Bn*	*Ws*	*Fh*	*La*	*Tr*	*Lr*	*Lia*	*Pp*	*Kt*
*Flustra foliacea *(*Ff*)	0	12	12	13	12	12	12	14	12	12
*Bugula neritina *(*Bn*)	12	0	12	12	10	9	14	13	9	9
*Watersipora subtorquata *(*Ws*)	12	12	0	13	14	14	14	15	14	14
*Flustrellidra hispida *(*Fh*)	13	12	13	0	13	13	14	15	13	13
*Loxosomella aloxiata *(*La*)	12	10	14	13	0	5	14	13	7	4
*Terebratulina retusa *(*Tr*)	12	9	14	13	5	0	15	13	4	2
*Laqueus rubellus *(*Lr*)	12	14	14	14	14	15	0	15	15	15
*Lingula anatina *(*Lia*)	14	13	15	15	13	13	15	0	14	13
*Phoronis psammophila *(*Pp*)	12	9	14	13	7	4	15	14	0	3
*Katharina tunicata *(*Kt*)	12	9	14	13	4	2	15	13	3	0

### Nucleotide composition and codon usage

There is a high variation in nucleotide composition of metazoan mitochondrial genomes. In our data set the variation of overall A+T content ranges from 51.4% (*Balanoglossus*) to 78.6% (*Heptathela*), with an average overall value equal to 66.5% (Table 2). The lowest values are found in Deuterostomia (average value 61.1%), the highest in Ecdysozoa (average value 69.7%). The average value of Lophotrochozoa (66.5%) and the overall A+T content of *Flustra foliacea *(66.5%) equal exactly the overall average (Table 2). The overall A+T content of *Flustra foliacea *corresponds with the A+T content of the partial mitochondrial genome of the phoronid *Phoronis psammophila *(66.6%) and is intermediate between that of the other sequenced cheilostomate ectoprocts *Bugula neritina *(70.0%) and *Watersipora subtorquata *(70.6%) and the entoprocts *Loxocorone allax *(73.4%) and *Loxosomella aloxiata *(70.6%) on the one hand, and that of the ctenostomate ectoproct *Flustrellidra hispida *(59.4%) and of the brachiopods (*Terebratulina retusa*: 57.2%; *Terebratalia transversa*: 59.1%; *Laqueus rubellus*: 58.3%) on the other. The high A+T content is also reflected in the individual protein-encoding genes (Table 3). It is especially high at third codon positions (72.4%).

**Table 2:** Nucleotide composition and AT-and CG-skews of mitochondrial genomes.

Taxon	Length (bp)	A	C	G	T	AT%	AT skew	GC skew
**Cnidaria**								
*Acropora tenuis*	18338	0.251	0.137	0.242	0.370	62.0%	-0.192	0.277
*Metridium senile*	17443	0.269	0.169	0.212	0.349	61.9%	-0.129	0.112
**Hemichordata**								
*Balanoglossus carnosus*	15708	0.251	0.314	0.171	0.264	51.4%	-0.026	-0.295
**Echinodermata**								
*Arbacia lixula*	15719	0.295	0.205	0.170	0.330	62.5%	-0.057	-0.091
*Florometra serratissima*	16005	0.264	0.116	0.156	0.464	72.8%	-0.274	0.149
**Chordata**								
*Homo sapiens*	16569	0.309	0.313	0.131	0.247	55.6%	0.112	-0.410
*Xenopus laevis*	17553	0.331	0.235	0.135	0.300	63.0%	0.049	-0.270
**Chaetognatha**								
*Paraspadella gotoi*	11423	0.394	0.147	0.125	0.334	72.8%	0.081	-0.082
*Spadella cephaloptera*	11905	0.364	0.182	0.167	0.286	65.0%	0.120	-0.044
**Priapulida**								
*Priapulus caudatus*	14919	0.303	0.144	0.165	0.388	69.1%	-0.123	0.068
**Nematoda**								
*Caenorhabditis elegans*	13794	0.314	0.089	0.149	0.448	76.2%	-0.175	0.253
*Trichinella spiralis*	16706	0.405	0.230	0.097	0.265	67.0%	0.209	-0.405
**Onychophora**								
*Epiperipatus biolleyi*	14411	0.320	0.086	0.173	0.421	74.1%	-0.135	0.334
**Arthropoda**								
*Limulus polyphemus*	14985	0.375	0.227	0.097	0.301	67.6%	0.111	-0.399
*Heptathela hangzhouensis*	14215	0.416	0.172	0.106	0.369	78.6%	0.059	-0.235
*Antrokoreana gracilipes*	14747	0.298	0.199	0.180	0.323	62.1%	-0.041	-0.049
*Lithobius forficatus*	15695	0.369	0.204	0.117	0.310	67.9%	0.087	-0.269
*Triops cancriformis*	15101	0.358	0.182	0.131	0.330	68.8%	0.041	-0.163
*Penaeus monodon*	15984	0.353	0.167	0.127	0.354	70.6%	-0.001	-0.136
*Atelura formicaria*	15205	0.348	0.246	0.130	0.276	62.4%	0.114	-0.308
*Tribolium castaneum*	15881	0.398	0.185	0.098	0.319	71.7%	0.109	-0.305
**Platyhelminthes**								
*Microcotyle sebastis*	14407	0.293	0.097	0.197	0.411	70.4%	-0.166	0.341
*Echinococcus granulosus*	13588	0.191	0.080	0.250	0.479	67.1%	-0.430	0.515
*Schistosoma japonicum*	14085	0.249	0.084	0.206	0.461	71.0%	-0.299	0.422
**Entoprocta**								
*Loxocorone allax*	14862	0.412	0.148	0.118	0.322	73.4%	0.123	-0.111
*Loxosomella aloxiata*	15323	0.392	0.163	0.131	0.314	70.6%	0.110	-0.108
**Ectoprocta**								
*Flustrellidra hispida*	13026	0.271	0.235	0.176	0.318	58.9%	-0.079	-0.142
*Watersipora subtorquata*	14144	0.364	0.163	0.131	0.342	70.6%	0.030	-0.108
*Bugula neritina*	15433	0.377	0.176	0.124	0.323	70.0%	0.078	-0.173
*Flustra foliacea*	16089	0.248	0.114	0.222	0.417	66.5%	-0.254	0.321
**Phoronida**								
*Phoronis psammophila *^a^	14018	0.334	0.168	0.166	0.332	66.6%	0.002	-0.005
**Brachiopoda**								
*Lingula anatina *^b^	28818	0.261	0.161	0.219	0.359	62.0%	-0.158	0.153
*Terebratulina retusa*	15451	0.295	0.277	0.151	0.277	57.2%	0.033	-0.294
*Laqueus rubellus*	14017	0.208	0.151	0.265	0.375	58.4%	-0.286	0.272
*Terebratalia transversa*	14291	0.199	0.134	0.275	0.392	59.1%	-0.328	0.344
**Nemertea**								
*Cephalothrix simula*	16296	0.275	0.102	0.148	0.474	74.9%	-0.266	0.182
*Lineus viridis*	15388	0.213	0.119	0.224	0.445	65.7%	-0.352	0.306
**Annelida**								
*Sipunculus nudus*	15502	0.268	0.297	0.161	0.274	54.2%	-0.013	-0.297
*Clymenella torquata*	15538	0.330	0.195	0.133	0.343	67.2%	-0.020	-0.188
*Urechis caupo*	15113	0.315	0.235	0.144	0.305	62.0%	0.016	-0.240
*Platynereis dumerilii*	15619	0.312	0.204	0.154	0.329	64.1%	-0.026	-0.141
*Lumbricus terrestris*	14998	0.298	0.225	0.158	0.318	61.6%	-0.031	-0.176
**Mollusca**								
*Katharina tunicata*	15532	0.314	0.119	0.186	0.380	69.4%	-0.095	0.220
*Graptacme eborea*	14492	0.370	0.132	0.127	0.371	74.1%	-0.002	-0.021
*Nautilus macromphalus*	16258	0.337	0.285	0.119	0.258	59.6%	0.133	-0.412
*Loligo bleekeri*	17211	0.388	0.195	0.092	0.325	71.3%	0.089	-0.358
*Octopus vulgaris*	15744	0.411	0.176	0.076	0.337	74.9%	0.099	-0.397
*Pupa strigosa*	14189	0.274	0.183	0.205	0.337	61.1%	-0.103	0.056
*Aplysia californica*	14117	0.286	0.154	0.182	0.377	66.3%	-0.137	0.085
*Biomphalaria glabrata*	13670	0.331	0.113	0.141	0.416	74.6%	-0.114	0.110

AT skew = (A%-T%)/(A%+T%); GC skew = (G%-C%)/(C%+G%); ^a ^partial; ^b ^repetitive

**Table 3:** Nucleotide composition and AT- and GC-skews of the mitochondrial protein-encoding and ribosomal RNA genes and the entire *Flustra foliacea *genome.

Gene	Proportion of nucleotides				AT%	AT skew	GC skew
				
	A	G	C	T			
*atp6*	0.213	0.225	0.123	0.439	65.2	-0.347	0.293
*atp8*	0.306	0.189	0.099	0.405	71.1	-0.139	0.313
*cox1*	0.227	0.219	0.135	0.419	64.6	-0.297	0.237
*cox2*	0.225	0.237	0.124	0.414	63.9	-0.296	0.313
*cox3*	0.196	0.266	0.110	0.426	62.2	-0.370	0.415
*cob*	0.225	0.214	0.130	0.430	65.5	-0.313	0.244
*nad1*	0.226	0.217	0.103	0.454	68.0	-0.335	0.356
*nad2*	0.246	0.217	0.104	0.434	68.0	-0.276	0.352
*nad3*	0.177	0.234	0.105	0.484	66.1	-0.464	0.381
*nad4*	0.214	0.219	0.106	0.462	67.6	-0.367	0.348
*nad4L*	0.212	0.242	0.072	0.474	68.6	-0.382	0.541
*nad5*	0.217	0.222	0.116	0.445	66.2	-0.344	0.314
*nad6*	0.187	0.224	0.085	0.503	69.0	-0.458	0.450
*rrnS*	0.336	0.215	0.142	0.306	64.2	0.047	0.204
*rrnL*	0.357	0.197	0.115	0.331	68.8	0.038	0.263
Entire genome	0.248	0.222	0.114	0.417	66.5	-0.254	0.321
Protein coding sequences	0.219	0.224	0.114	0.442	66.1	-0.337	0.325
1st codon position	0.27	0.257	0.117	0.358	62.8	-0.140	0.374
2nd codon position	0.169	0.183	0.186	0.462	63.1	-0.464	-0.008
3rd codon position	0.218	0.233	0.042	0.506	72.4	-0.398	0.695

AT skew = (A%-T%)/(A%+T%); GC skew = (G%-C%)/(C%+G%)

There is a high variation in AT- and GC-skews in metazoan mitochondrial genomes. In our data set AT-skews range from -0.430 (*Echinococcus*) to 0.209 (*Trichinella*) (Table 2). The range of GC-skews extends from -0.412 (*Nautilus*) to 0.515 (*Echinococcus*) (Table 2). Compared with other ectoprocts, *Flustra foliacea *is characterized by high AT- and GC-skews (Table 2). Among lophophorates, similarly high AT- and GC-skews have been found only in some brachiopods (*Laqueus*, *Terebratalia*). Nine genes of *Flustra *(*atp6*, *cox3*, *cob*, *nad1*, *nad3*, *nad4*, na*d*4L, *nad5*, *nad6*) have an AT-skew higher than 0.3 and ten genes (*atp8*, *cox2*, *cox3*, *nad1*, *nad2*, *nad3*, *nad4*, *nad4L*, *nad5*, *nad6*) have a GC-skew higher than 0.3 (Table 3). The GC-skew is positive for all 13 protein-encoding and the two ribosomal RNA genes, whereas the AT-skew is positive for all 13 protein-encoding genes, but negative for the two ribosomal RNA genes in *Flustra foliacea *mitochondria (Table 3).

**Figure 4:**
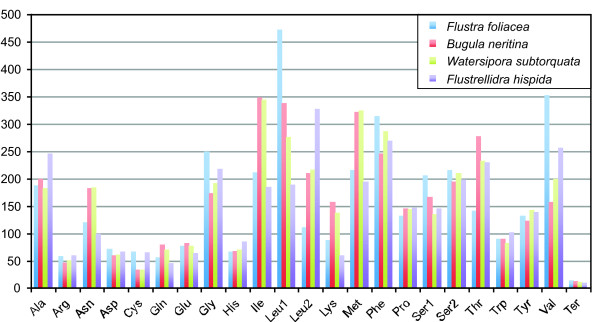
**Comparison of codon family usage in ectoproct mtDNAs**.

There are 3,605 codons for all protein coding genes in the mitochondrial genome of *Flustra*. The total number of codons is similar in the cheilostomate ectoprocts (3,605-3,668), whereas it was distinctly lower in the ctenostomate ectoproct *Flustrellidra *(3,356). Corresponding to the high percentage of T in the mitochondrial genome of *Flustra*, there is a bias towards T-rich codons (Additional file 1). The most frequently used codons are UUU (296 times) for phenylalanine, UUA (239) and UUG (231) for leucine, AUU (196) for isoleucine, and GUU (185) for valine. The most often used codon families in *Flustra *are Leu1, Val, Phe, Gly and Ser2. The least represented codon families are His, Gln, Arg, Cys and the termination codons. Compared with other ectoprocts, *Flustra *has a higher Leu1 and Val and a lower Leu2 and Thr codon usage (Figure 4, Additional file 1).

Four-fold degenerate codon usage is A/T biased in the third position, and T is the preferred nucleotide (Additional file 1). T is also the preferred nucleotide in two-fold degenerate codons ending in T or C. The codon usage is less biased in two-fold degenerate codons ending in A or G, with A predominating in Leu1, Lys and Met, and G predominating in Gln, Glu, Trp and the termination codons.

### Phylogenetic analyses of the relationships of the lophophorate lineages

The major results of the phylogenetic analyses of the nucleotide as well as the amino acid sequences of the mitochondrial protein-encoding genes concerning the relationships of the lophophorate lineages, ectoprocts, brachiopods and phoronids, are summarized in Table 4.

**Table 4:** Phylogenetic relationships of ectoprocts, brachiopods and phoronids according to different phylogenetic analyses (only sister group relationships with one other phylum; more complex relationships are not considered).

Method	Data set	Tree Figure	Ectoprocta+Phoronida	Ectoprocta+Entoprocta	Ectoprocta+Annelida	Ectoprocta+Gastropoda	Brachiopoda+Annelida	Phoronida+Nemertea	Phoronida+Entoprocta
Maximum-likelihood (MtZoa+F model)	Amino acid data set, with *Lingula*	Additional file 2							<50
Maximum-likelihood (GTR model)	Nucleotide data set	Additional file 3				<50	86	<50	
Maximum-likelihood (GTR model)	Nucleotide data set (Gblocks edited)	Additional file 4					<50		
Maximum-likelihood (GTR model)	Nucleotide data set (direct nucleotide alignment)	Additional file 5					99		
Maximum-likelihood (MtZoa+F model)	Amino acid data set	Additional file 6					52		52
Maximum-likelihood (MtZoa+F model)	Amino acid data set (Gblocks edited)	Additional file 7				<50		<50	
Maximum-likelihood (GTR model)	1^st ^and 2^nd ^codon positions	5B				<50	<50	<50	
nhPhyML	Nucleotide data set; starting tree GTR tree	Additional file 8				x		x	
nhPhyML	Nucleotide data set; starting tree CAT tree	Additional file 9	x						
Bayesian (CAT model)	Amino acid data set	5A			0.84				
Bayesian (CAT model)	Amino acid data set; 10 taxa with the most strongly differing amino acid composition excluded	Additional file 11			0.78				0.58
Maximum-likelihood (MtZoa+F model)	Amino acid data set; 10 taxa with the most strongly differing amino acid composition excluded	Additional file 12		<50			<50		
Bayesian (CAT model)	Amino acid data set recoded using 9 minmax chi-squared bins'	Additional file 14			0.92				
Maximum-likelihood (MULTIGAMMA model)	Amino acid data set recoded using 9 minmax chi-squared bins	Additional file 15					60		
Bayesian (CAT model)	Amino acid data set recoded using 6 minmax chi-squared bins	Additional file 16					0.96		
Maximum-likelihood (MULTIGAMMA model)	Amino acid data set recoded 6 minmax chi-squared bins	Additional file 17					<50		<50
Bayesian (CAT model)	Amino acid data set recoded using Dayhoff groups	Additional file 18							
Maximum-likelihood (MULTIGAMMA model)	Amino acid data set recoded Dayhoff groups	Additional file 19							<50
Bayesian (CAT+BP model)	Amino acid data set	Additional file 20			0.63				
Maximum-likelihood (GTR model)	Nucleotide data set, 20% of the alignment positions with highest sitewise rates removed	Additional file 21					98		
Maximum-likelihood (MtZoa+F model)	Amino acid data set; 10% of the alignment positions with highest sitewise rates removed	Additional file 22					<50		58

Unless noted otherwise, the analyses are based on alignments edited with ALISCORE and the nucleotide alignments are derived from the amino acid alignments. If a group is monophyletic, the posterior probability respectively the bootstrap support is given.

Initially, we included all completely sequenced mitochondrial genomes of lophophorate lineages in the phylogenetic analysis (Additional file 2). However, the mitochondrial genes of the brachiopod *Lingula *are generally longer and deviate considerably in sequence from their orthologs in other animals [51]. Therefore, these sequences introduced ambiguities into the alignments. Thus, we excluded this taxon from all further phylogenetic analyses.

The newly sequenced cheilostomate ectoproct *Flustra *clusters in all analyses with the two other included cheilostomate ectoprocts *Bugula *and *Watersipora*. Ectoprocta is also monophyletic in all analyses. In the majority of the analyses *Flustra *is sister group to *Bugula*. Only in some analyses *Bugula *is sister taxon to *Watersipora *instead. A closer relationship of *Bugula *to *Flustra *than to *Watersipora *(or other Lepraliomorpha, to which *Watersipora *belongs) is also supported by the presence of long intergenic sequences and the structure of some tRNAs in these taxa (see above) and by phylogenetic analyses based on 18S rDNA, 28S rDNA and *cox1 *sequences [52].

The lophophorate lineages are usually more closely related to trochozoan phyla than to deuterostomes or ecdysozoans confirming the Lophotrochozoa hypothesis. Only in a few of the analyses, ectoprocts cluster with a long-branch group including platyhelminths, nematodes and chaetognaths. However, the sister group relationships of the lophophorate lineages within Lophotrochozoa differ strongly depending on the data set, method and evolutionary model (Table 4). The different sister group relationships are not strongly supported by the data and may be affected by stochastic as well as systematic errors. Surprisingly, a sister group relationship between Ectoprocta and Brachiopoda as reconstructed in several other analyses of mitochondrial sequences [19,37-39] was not recovered in any of our analyses. The same applies to the previously proposed sister group relationship between Ectoprocta and Chaetognatha [19,37,39,49]. These vagaries indicate that there is no robust phylogenetic signal for such relationships in the mitochondrial sequences.

In the maximum likelihood tree (Additional file 3) calculated based on the nucleotide alignment derived from the amino acid alignment and edited with ALISCORE [53,54] comprising 12,648 positions of 49 taxa using the GTR model implemented in RAxML, a sister group relationship between brachiopods and annelids is comparatively well-supported (86% bootstrap value). In this as well as in several of the following analyses platyhelminths, nematodes and chaetognaths, all of them characterized by high substitution rates, form a monophylum, so that neither Ecdysozoa nor Lophotrochozoa are monophyletic. Such long branch artefacts have also been found in most other phylogenetic analyses of mitochondrial nucleotide and amino acid sequences (e.g., [32,38,39,55]). The topology of the maximum likelihood tree based on the nucleotide alignment edited with Gblocks [56] (including 6,839 positions) differs from that based on the alignment edited with ALISCORE only with regard to nodes that are not well supported in any of the trees (Additional file 4). The topology of the maximum likelihood tree based on a direct nucleotide alignment (edited with ALISCORE; including 12,648 positions; Additional file 5) does not differ from that based on the nucleotide alignment derived from the amino acid alignment in any strongly supported nodes.

**Figure 5:**
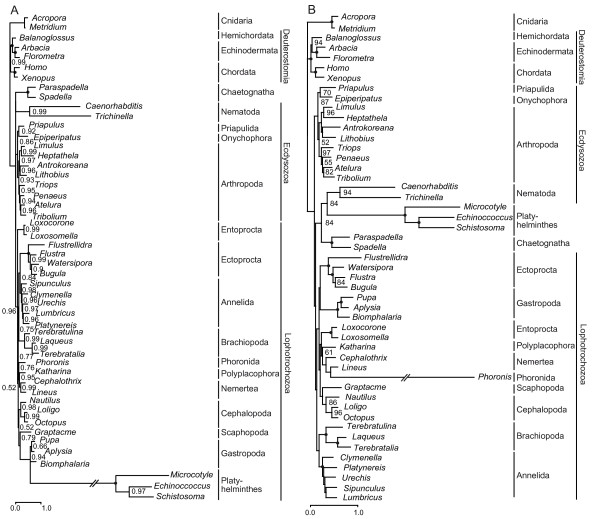
**Metazoan phylogeny based on mitochondrial sequences of 49 taxa**. (*A*) Bayesian inference reconstructions calculated with the CAT model based on 2,729 amino acid positions. Bayesian posterior probabilities are shown to the right of the nodes; posterior probabilities equal to 1.0 are indicated by black circles. (*B*) Maximum likelihood tree calculated with the GTR model based on 7,537 nucleotides from first and second codon positions. Bootstrap support values larger than 50% are shown to the right of the nodes; 100% bootstrap values are indicated by black circles.

In the Bayesian inference tree based on the mitochondrial amino acid data set edited with ALISCORE [53,54] comprising 2,729 positions of 49 taxa calculated with the CAT model implemented in PhyloBayes (Figure 5A), the long-branch group is broken up and Lophotrochozoa including Platyhelminthes form a well-supported monophylum (posterior probability 0.96). The maximum likelihood analysis of this data set with the MtZoa+F model (Additional file 6) resulted again in a long-branch attraction of platyhelminths, nematodes and chaetognaths. The monophyly of most of the lophotrochozoan phyla with the exception of the molluscs is strongly supported in both analyses, but the relationships between these phyla remains unresolved. The maximum likelihood tree based on the amino acid sequences edited with Gblocks [56] (Additional file 7) does not differ from that edited with ALISCORE in any strongly supported nodes. In the Bayesian inference tree ectoprocts are sister group of annelids (posterior probability 0.84), and brachiopods are sister group of this monophylum (0.75). Phoronida is sister group of a clade consisting of Nemertea and Polyplacophora (0.76). In contrast, according to the maximum likelihood tree ectoprocts are sister group to the long-branch group consisting of nematodes, platyhelminths and chaetognaths. Brachiopods are sister group of annelids (52% bootstrap probability) and phoronids are sister group of entoprocts (52%).

### Evaluation of compositional heterogeneity of mitochondrial nucleotide sequences and phylogenetic analyses accounting for it

A chi-square test indicates that the nucleotide composition of the used mitochondrial nucleotide sequences is significantly heterogeneous between lineages (chi-square = 23,209 (df = 144), P = 0.000). This is confirmed by the matched-pairs tests of symmetry, according to which 99.6% of the pairwise comparisons show significant (P < 0.050) heterogeneity. Although the nucleotide composition is heterogeneous at all codon positions, it is less pronounced at the first (chi-square = 5,814 (df = 144), P = 0.000; 97.5% significantly heterogeneous pairs) and second (chi-square = 2,990 (df = 144), P = 0.000; 90.7% significantly heterogeneous pairs) than at the third codon positions (chi-square 24,521 (df = 144), P = 0.000; 99.3% significantly heterogeneous pairs).

A maximum likelihood analysis based on the first and second codon positions only resulted in a reduction of the support for a brachiopod-annelid sister group relationship (Figure 5B), indicating that this grouping might be an artefact resulting from compositional bias.

Alternatively, we accounted for the compositional heterogeneity in the nucleotide sequences by using the nonstationary model implemented in nhPhyML-Discrete. This analysis requires a starting tree, for which we used the maximum likelihood tree obtained with the nucleotide data set and the GTR model as well as the Bayesian inference tree based on the amino acid sequences obtained with the CAT model (see below). The two analyses resulted in strongly different topologies (Additional file 8, 9). The tree obtained with the starting tree based on the nucleotide data set and the GTR model had a slightly higher likelihood (loglk = -375,007) than the tree obtained with the starting tree based on the amino acid data set (loglk = -375,103). In the latter platyhelminths are included in Lophotrochozoa and phoronids are sister group of ectoprocts, whereas in the former platyhelminths are the sister group of nematodes and *Phoronis *is nested in Nemertea.

### Evaluation of compositional heterogeneity of mitochondrial amino acid sequences and phylogenetic analyses accounting for it

We evaluated the potential influence of compositional heterogeneity in the amino acid data set on the phylogenetic analyses by a posterior predictive test based on the PhyloBayes analysis of the complete data set (Table 5; Additional file 10). This test indicates that the assumption of compositional homogeneity made by most models for amino acid sequence evolution is strongly violated in the mitochondrial amino acid data (global *Z *score 8.657, Table 5; Additional file 10). The test statistic for individual taxa indicates that the amino acid composition of 40 of the 49 taxa is significantly deviating. The compositional bias is much stronger than that found in a nuclear ribosomal protein data set [34]. Thus, there might be artifacts resulting from compositional bias in the trees calculated with the usual evolutionary models.

**Table 5:** Results of posterior predictive tests indicating the ability of different approaches to reduce compositional bias in mitochondrial amino acid data sets.

Approach	Remaining taxa	*Z *score	*p *value	Number of taxa with significantly deviating amino acid composition
Original data set	49	8.657	0.000	40
Exclusion of the 10 taxa with the most strongly differing amino acid composition	39	7.308	0.000	32
Recoding using 9 minmax chi-squared bins	49	8.690	0.003	38
Recoding using 6 minmax chi-squared bins	49	7.196	0.005	21
Recoding using Dayhoff groups	49	11.285	0.000	30

One approach to reduce the compositional heterogeneity of the data set is the exclusion of taxa with strongly deviating amino acid composition. Obviously, not all 40 taxa with significantly deviating amino acid composition can be removed from the phylogenetic analysis. After excluding the ten taxa with the most strongly deviating amino acid composition from the calculations (Additional files 11, 12), the CAT model is still significantly violated (global *Z *score 7.308; Table 5; Additional file 10) and the test statistic for individual taxa indicates that the amino acid composition of 32 taxa is significantly deviating. Remarkably, Ectoprocta and Entoprocta form a monophylum, Bryozoa, in the maximum likelihood tree based on the reduced data set as in some analyses of phylogenomic [26,27,29-34] and rDNA data sets [14-16], albeit with no nodal support (Additional file 12).

Another approach for reducing compositional heterogeneity is recoding of amino acids in bins. We determined bins that minimize compositional heterogeneity with the minmax method described by Susko and Roger [57]. Whereas the minimum *P *values for 10 or more bins are smaller than 0.05 (Additional file 13), the minimum *P *value for 9 minmax chi-squared bins (D, PV, AIMSY, GFT, L, NH, W, RCQK, E) is 0.112, which indicates that compositional homogeneity cannot be rejected for these bins according to the chi-square test. However, a posterior predictive test shows that the compositional heterogeneity has not been reduced (global *Z *score 8.690) and that the CAT model is still significantly violated (Table 5; Additional file 10) if the amino acid sequences of the mitochondrial proteins were recoded using these bins. This contradiction between the results of the chi-square test and the posterior predictive test might be explained by the fact that the chi-square test does not consider correlation due to relatedness of the taxa on a tree or by the biasing effect of invariable sites on this test [58,59]. A reduction of the categories to 6 minmax chi-squared bins resulted only in a minor reduction of the compositional heterogeneity (global *Z *score 7.196; Table 5; Additional file 10) despite the minimum *P *value for 6 bins (GFTW, AHILMSY, NPV, E, D, RCQK) being 0.21 according to the chi-square test.

Alternatively, we recoded the amino acid data into the six groups of amino acids (AGPST, C, DENQ, FWY, HKR, ILMV) that tend to replace one another [60]. A posterior predictive test showed that the compositional heterogeneity even increased (global *Z *score 11.285) compared to the unrecoded data set (Table 5; Additional file 10).

The phylogenetic analyses of recoded data sets (Additional files 14, 15, 16, 17, 18, 19) yielded again contradictory results concerning the relationships of the lophophorate lineages (Table 4). None of the possible relationships of the lophophorate lineages is strongly supported.

We analysed the amino acid sequences also with a non-stationary model of sequence evolution by performing a Bayesian analysis with the CAT-BP model as implemented in the program nhPhyloBayes [61]. We started 16 chains with the mitochondrial amino acid data set. The mean number of breakpoints *N*, at which the amino acid composition changes, varied between 34 and 47. Because the prior on *N *used in the CAT-BP model is conservative, an *N *as high as observed in our analysis confirms that there is compositional bias in the data. The high number of breakpoints reflects the result of the posterior predictive test that 40 taxa belonging to several different clades have amino acid compositions that significantly deviate from the assumptions of the CAT model (Additional file 10). Despite almost nine weeks of calculation for each chain on a 2.8 GHz processor no convergence of the chains was achieved. A consensus of all chains is shown for illustrative purposes (Additional file 20). Lophotrochozoa including Platyhelminthes is monophyletic, but the relationships between lophotrochozoan phyla are largely unresolved.

### Phylogenetic analyses accounting for saturation

Finally, we tried to mitigate saturation and long-branch-attraction problems by excluding fast evolving sites. We removed 20% of the positions with high rates from the nucleotide alignment (10,118 nucleotides remaining) and 10% of the amino acid alignment positions (2,456 amino acid remaining). Despite the exclusion of the fastest evolving sites, the long-branch group including platyhelminths, nematodes and chaetognaths could not be broken up (Additional file 21, 22) and the relationships between the lophotrochozoan phyla could not be resolved more robustly. However, there is strong support (98% bootstrap probability) for a sister group relation between brachiopods and annelids in the tree based on the nucleotide data set.

## Conclusions

Altogether, the results obtained in the phylogenetic analyses of the mitochondrial nucleotide and amino acid sequences are contradictory and weakly supported by the data (Table 4). Most of the results concerning the phylogenetic relationships of the lophophorate lineages are in strong contrast to the results of recent phylogenomic analyses [26,27,29-31,33,34] and phylogenetic analyses of nuclear rDNA [14-16] that support the monophyly of Bryozoa (= Polyzoa) including Ectoprocta and Entoprocta as well as the monophyly of Brachiozoa including Brachiopoda and Phoronida. Jang and Hwang [38] showed that a topology test based on mitochondrial amino acid data rejects both, Brachiozoa and Bryozoa. Thus, the differences between the phylogenetic results based on mitochondrial data and the phylogenomic analysis based mainly or exclusively on nuclear data cannot be attributed to stochastic errors alone. The posterior predictive tests indicate that the phylogenetic analyses of the mitochondrial amino acid sequences are strongly affected by compositional bias, a systematic error source that is not taken into account by topology tests. Thus, the apparent contradiction between the phylogenetic results based on mitochondrial amino acid data and the phylogenomic analyses may be due to compositional bias. This is supported by the results of the approaches to reduce compositional heterogeneity in the data sets respectively the analyses with non-stationary models (Table 4). Although Bryozoa including Ectoprocta and Entoprocta were rejected in the topology tests performed by Jang and Hwang [38] based on mitochondrial amino acid data, Bryozoa was found in our maximum likelihood analysis with the MtZoa+F model with the 39 taxa set, albeit with no nodal support (Additional file 12).

Phylogenetic analyses of nuclear protein sequence data of Metazoa are also affected by compositional bias [34,62]. However, none of several approaches accounting for this bias supported a sister group relationship between Ectoprocta and Brachiopoda or between Phoronida and Entoprocta [34] as did some of the phylogenetic analyses of mitochondrial data ([19,37-39]; Table 4).

The weak support for relationships between phyla in the analyses based on the mitochondrial data (Table 4) indicates that the information content of the mitochondrial sequence data set, which is almost one magnitude smaller than current phylogenomic data sets, is insufficient for a robust resolution of the divergences of the lophotrochozoan phyla (see also [19,38]). In addition, the strong compositional bias in the mitochondrial data (Table 5; Additional file 10) complicates phylogenetic analyses of these data. The high variability of the gene order in some lophotrochozoan phyla like ectoprocts, brachiopods or molluscs undoes the hope that this character set may help to disentangle the relationships between lophotrochozoan phyla. With current methods and evolutionary models mitochondrial genome data can contribute little to resolving the relationships of the lophotrochozoan phyla.

However, our study revealed several rare genomic changes like the loss of the DHU arm and changes of the anticodon sequence of tRNAs and the evolution of long intergenic sequences, that may be helpful for reconstructing ectoproct phylogeny more robustly in future studies.

## Methods

### DNA extraction

A sample of *Flustra foliacea *(Ectoprocta, Gymnolaemata) was obtained from the Biologische Anstalt Helgoland (Germany) and conserved at -70°C. Total genomic DNA was extracted with the QIAamp DNA Mini kit (Qiagen, Hilden, Germany) following the manufacturer's instructions for tissue.

### PCR amplification, cloning and sequencing

Mitochondrial sequence fragments of the genes *nad1*, *nad2*, *nad3*, *nad4*, *nad5*, *nad6*, *cob*, *cox1*, *cox2*, *cox3*, *atp6*, and *rrnS *from an EST library of *Flustra foliacea *(Hausdorf et al., 2007) were used to design specific primers (Additional file 23). The complete mitochondrial genome of *Flustra foliacea *was amplified with these primers. All PCRs were done in an Eppendorf Mastercycler Gradient thermocycler. PCRs were carried out in 50 μl volumes (33.75 μl water, 10 μl 5× amplification buffer (Promega, Mannheim, Germany), 2 μl MgCl_2 _solution (25 mM), 0.25 μl Taq polymerase (5 U/μl), 1 μl dNTP mixture (25 mM each), 1 μl template DNA, 2 μl primer mixture (10 μM each)) using GoTaq polymerase (Promega, Mannheim, Germany). To minimize replication errors, proof-reading Pwo polymerase (Roche, Mannheim, Germany) was added to the reaction mix. Cycling conditions were as follows: 94°C for 120 s for initial denaturation, 35 cycles of 94°C for 30 s, 45-55°C for 30 s, 72°C for 180 s, followed by 72°C for 420 s for final elongation. If amplifications were not successful, DNA fragments were amplified with the PCR Extender System (5Prime, Darmstadt, Germany) in 50 μl volumes (38.1 μl water, 5 μl 10× tuning buffer, 0.4 μl PCR Extender Polymerase Mix, 2.5 μl dNTP mixture, 2 μl template DNA, 2 μl primer mixture (10 μM each)) under the following long PCR conditions: 93°C for 180 s for initial denaturation, 10 cycles of 93°C for 15 s, 55-62°C for 30 s, 68°C for 900 s, 20 cycles of 93°C for 15 s; 55-62°C for 30 s, 68°C for 900 s plus 20 s for each cycle. PCR fragments were excised from agarose gel and purified with the NucleoSpin Extract II kit (Macherey-Nagel, Düren, Germany). Dependent on the band intensity on the agarose gel, DNA was eluted in 20-50 μl elution buffer or ddH_2_O and stored at -20°C. Each purified fragment was ligated into the pCR2.1-TOPO cloning vector (Invitrogen, Karlsruhe, Germany) and transformed into *Escherichia coli *TOP10 cells (Invitrogen, Karlsruhe, Germany). Clones containing inserts of the correct size were sequenced on an automatic capillary sequencer. Large inserts were sequenced by primer walking (sequences available on request).

### Sequence assembly and annotation

Sequence assembly was done with SeqMan (DNASTAR, Madison, WI). The average coverage of the genome by sequenced clones or EST contigs was 2.4×. Protein-encoding and ribosomal RNA genes were identified by BLAST (blastn, tblastx) searches of NCBI databases and by using the MITOS WebServer BETA (http://bloodymary.bioinf.uni-leipzig.de/mitos/index.py ). Start and end positions of rRNA genes and MNCR were determined by boundaries of adjacent genes. The tRNA genes were detected via class-specific co-variance models using the MITOS WebServer BETA. Complementarily, tRNAscan-SE [63] and ARWEN [64] were used. The sequence data was deposited in GenBank with the accession number JQ061319. We used CRex [50] to analyse gene order data. GC- and AT-skew was calculated by using the formula of Perna and Kocher [65].

### Alignment

For phylogenetic analyses, we focused the taxon sampling (Additional file 24) on lophotrochozoan taxa. We assembled complete or nearly complete mitochondrial genomes available from members of the phyla Ectoprocta (4 species), Brachiopoda (4), Phoronida (1), Entoprocta (2), Nemertea (2), Chaetognatha (2) and selected 8 representative mitochondrial genomes from molluscs, 5 from annelids and 3 from platyhelminths. We added 12 ecdysozoan and 5 deuterostome species as well as 2 cnidarian taxa as outgroups.

The amino acid sequences of the mitochondrial protein-encoding genes of the selected taxa were individually aligned by the L-INS-i algorithm implemented in MAFFT [66,67]. Because it is preferable to take the amino acid level into account during alignment of protein-coding DNA, the aligned amino acid sequences were used as a scaffold for constructing the corresponding nucleotide sequence alignment using RevTrans 1.4 [68]. For comparison, the nucleotide sequences were aligned directly. We identified randomly similar sections in each gene alignment with ALISCORE [53,54] on the nucleotide and amino acid level using default settings and maximal number of pairwise comparisons. In total, 15% of originally 14,968 nucleotide positions and 39% of originally 4,452 amino acid positions were excluded using ALICUT (http://www.utilities.zfmk.de) to increase the signal-to-noise ratio. The final alignments, spanning 12,648 nucleotide respectively 2,729 amino acid positions, were attained by concatenating all processed alignments. Alternatively to the ALISCORE evaluation of the sequences, we used Gblocks [56] with low stringency parameters (minimum block length 5; allowed gap positions with half) for eliminating poorly aligned positions and divergent regions resulting in concatenated alignments spanning 6,839 nucleotide respectively 1,862 amino acid positions. The final alignments have been deposited at TreeBASE and can be accessed at http://purl.org/phylo/treebase/phylows/study/TB2:S10996. Alignments with reduced taxa sets were obtained by removing taxa from the complete alignments. Unless otherwise noted, the alignments edited with ALISCORE were used.

### Phylogenetic analyses and evaluation of model violation caused by compositional heterogeneity

We checked the homogeneity of nucleotide frequencies across taxa using the chi-square test implemented in PAUP* 4.0 beta 10 [69]. However, this test ignores correlation resulting from phylogenetic structure. Therefore, we also measured the probability that the base composition of two sequences is homogeneous for each pair of sequences using the matched-pairs test of symmetry as implemented in SeqVis version 1.4 [70].

We performed maximum likelihood analyses using a parallel Pthreads-based version [71] of RAxML, version 7.2.8 [72]. We used the GTR model for nucleotide sequences, the MtZoa+F model [73] for amino acid sequences, and the MULTIGAMMA model for recoded amino acid data (see below). Using a modified perl script for model selection based on likelihood calculations with RAxML (available from http://icwww.epfl.ch/~stamatak/index-Dateien/software/ProteinModelSelection.pl), the MtZoa+F model [73] was selected for amino acid sequences. Rate heterogeneity among sites was modelled using the gamma model. Confidence values for edges of the maximum likelihood tree were computed by rapid bootstrapping [74] (100 replications).

We performed Bayesian inference analyses of the amino acid sequences with the CAT model that adjusts for site-specific amino acid frequencies [75] as implemented in PhyloBayes version 3.2f (http://megasun.bch.umontreal.ca/People/lartillot/www/download.html). Eight independent chains were run for each analysis. The number of points of each chain, the number of points that were discarded as burn-in, and the largest discrepancy observed across all bipartitions (maxdiff) are listed in Additional file 25. Taking every tenth sampled tree, a 50%-majority rule consensus tree was computed using all chains.

We evaluated in how far the assumptions of the CAT model are violated by using posterior predictive tests. In posterior predictive tests the observed value of a given test statistic on the original data is compared with the distribution of the test statistic on data replicates simulated under the reference model using parameter values drawn from the posterior distribution (every tenth sampled tree). The reference model is rejected for that statistic if the observed value of the test statistic deviates significantly. We used two test statistics measuring compositional heterogeneity implemented in PhyloBayes. One measures the compositional deviation of each taxon by summing the absolute differences between the taxon-specific and global empirical frequencies over the 20 amino acids. This test statistic indicates which taxa deviate significantly, but raises a multiple-testing issue. Alternatively, the maximum deviation across taxa was used as a global statistic.

### Approaches for reducing the potential impact of compositional bias

Because the third codon positions show the strongest compositional heterogeneity (see results) and because these positions become saturated first because of their higher substitution rates, we tried to reduce the potential impact of systematic errors on phylogenetic inference by excluding the third codon positions from the nucleotide data set.

We applied two approaches to reduce compositional heterogeneity in the amino acid data set. First, we excluded the taxa with the most strongly deviating amino acid composition as indicated by the posterior predictive test and repeated the Bayesian inference analysis as described. Secondly, we recoded the amino acid data into groups. Susko and Roger [57] developed an algorithm for constructing bins of amino acids in order to minimize compositional heterogeneity for a given alignment by minimizing the maximum chi-squared statistic for a taxon of the data set. We used the program minmax-chisq (http://www.mathstat.dal.ca/tsusko/software.cgi) to obtain these minmax chi-squared bins for the mitochondrial amino acid data set. In order to lose as little information as possible, we chose the largest number of bins for which the minimum *P *value is larger than 0.05, which indicates that compositional homogeneity cannot be rejected for this set of bins according to the chi-square test. Alternatively, we recoded the amino acid data into the six groups of amino acids (AGPST, C, DENQ, FWY, HKR, ILMV) that tend to replace one another [60].

As alternative to the approaches for reducing compositional heterogeneity in the data set, we used nonstationary models of evolution in phylogenetic inference analyses. We analysed the nucleotide data set using the nonstationary model of evolution developed by Galtier and Gouy [76] as implemented in nhPhyML-Discrete [77], limited to 3 base content frequency categories and with 8 categories for a discrete gamma model of among-site rate variation. Based on the amino acid data set, we performed a Bayesian analysis with the CAT-BP model [61] as implemented in nhPhyloBayes (http://www.lirmm.fr/mab/blanquart/), which accounts for compositional heterogeneity between lineages by introducing breakpoints along the branches of the phylogeny at which the amino acid composition is allowed to change. Sixteen independent chains were run for 10,000 points. Stationarity of the posterior probabilities of all chains were reached during the first 2,000 points. Thus, 2,000 points were discarded as burn-in for all chains. Taking every tenth sampled tree, a 50%-majority rule consensus tree was computed.

### Approaches for reducing the potential impact of saturation and long-branch attraction

To mitigate the potential impact of saturation and long-branch attraction, we excluded the fastest evolving sites as determined by Treefinder, version of October 2008 [78,79]. An appropriate model for nucleotide respectively protein evolution was determined with the 'propose model' option of Treefinder based on the Akaike Information Criterion with a correction term for small sample size. According to this criterion the GTR model with gamma-distributed rates was chosen for the nucleotide data set and a mixed model that is a linear combination of 14 empirical models of protein evolution and considering among-site rate variation with a five-category discrete gamma-distribution for rates was chosen for the amino acid data set. With the data sets and these models maximum likelihood trees were calculated with Treefinder. Finally, sitewise rates were calculated with the data sets, the models and the trees as input.

## Abbreviations

A: adenine; *atp6 *and *atp8*: genes encoding ATPase subunits 6 and 8; bp: base pairs; C: cytosine; *cox1-3*: genes encoding cytochrome C oxidase subunits I-III; *cob*: gene encoding cytochrome b; G: guanine; MNCR, major non-coding region; *nad1-6 *and *nad4L*: genes encoding NADH dehydrogenase subunits 1-6 and 4L; *rrnS *and *rrnL*: genes encoding small (12S) and large (16S) rRNAs; T: thymine.

## Authors' contributions

MN extracted the DNA and carried out the amplifications, the sequencing and the sequence alignments and performed the phylogenetic analyses. MH provided EST sequences. IB and MN made the sequence assembly. BH and IB designed the study. BH drafted the manuscript. All authors contributed to, read and approved the final manuscript.

## Supplementary Material

Additional file 1 **Codon usage pattern of the mitochondrial protein-encoding genes in ectoprocts**. Click here for file

Additional file 2 **Maximum likelihood tree calculated with the MtZoa+F model based on 2,729 amino acid positions (ALISCORE edited) of 50 metazoan taxa**. Bootstrap support values larger than 50% are shown to the right of the nodes; 100% bootstrap values are indicated by black circles. Click here for file

Additional file 3 **Maximum likelihood tree calculated with the GTR model based on 12,648 nucleotide positions (ALISCORE edited) of 49 metazoan taxa**. Bootstrap support values larger than 50% are shown to the right of the nodes; 100% bootstrap values are indicated by black circles. Click here for file

Additional file 4 **Maximum likelihood tree calculated with the GTR model based on 6,839 nucleotide positions (Gblocks edited) of 49 metazoan taxa**. Bootstrap support values larger than 50% are shown to the right of the nodes; 100% bootstrap values are indicated by black circles. Click here for file

Additional file 5 **Maximum likelihood tree calculated with the GTR model based on 12,648 nucleotide positions (direct nucleotide alignment; ALISCORE edited) of 49 metazoan taxa**. Bootstrap support values larger than 50% are shown to the right of the nodes; 100% bootstrap values are indicated by black circles. Click here for file

Additional file 6 **Maximum likelihood tree calculated with the MtZoa+F model based on 2,729 amino acid positions (ALISCORE edited) of 49 metazoan taxa**. Bootstrap support values larger than 50% are shown to the right of the nodes; 100% bootstrap values are indicated by black circles. Click here for file

Additional file 7 **Maximum likelihood tree calculated with the MtZoa+F model based on 1,862 amino acid positions (Gblocks edited) of 49 metazoan taxa**. Bootstrap support values larger than 50% are shown to the right of the nodes; 100% bootstrap values are indicated by black circles. Click here for file

Additional file 8 **Maximum likelihood tree calculated with the nonstationary model implemented in nhPhyML-Discrete based on 10,629 nucleotide positions (ALISCORE edited) of 49 metazoan taxa**. The maximum likelihood tree obtained with the nucleotide data set and the GTR model (Additional file 3) was used as starting tree. Click here for file

Additional file 9 **Maximum likelihood tree calculated with the nonstationary model implemented in nhPhyML-Discrete based on 10,629 nucleotide positions (ALISCORE edited) of 49 metazoan taxa**. The Bayesian inference tree based on the amino acid sequences obtained with the CAT model (Figure 5A) was used as starting tree. Click here for file

Additional file 10 Results of the posterior predictive tests concerning compositional heterogeneity in differently modified concatenated alignments of mitochondrial proteins. Significant values of the *Z *scores are marked by *. Click here for file

Additional file 11 **Bayesian inference reconstruction with the CAT model based on 2,623 amino acid positions (ALISCORE edited) of 39 metazoan taxa (excluding the 10 taxa with the most significantly deviating amino acid composition)**. Bayesian posterior probabilities are shown to the right of the nodes; posterior probabilities equal to 1.0 are indicated by black circles. Click here for file

Additional file 12 **Maximum likelihood tree calculated with the MtZoa+F model based on 2,623 amino acid positions (ALISCORE edited) of 39 metazoan taxa (excluding the ten taxa with the most significantly deviating amino acid composition)**. Bootstrap support values larger than 50% are shown to the right of the nodes; 100% bootstrap values are indicated by black circles. Click here for file

Additional file 13 **Minimum *P *values calculated with a chi-squared compositional heterogeneity test for maximum chi-square statistic bins based on the mitochondrial amino acid data set**. Click here for file

Additional file 14 **Bayesian inference reconstruction with the CAT model based on 2,729 amino acid positions (ALISCORE edited) of 49 metazoan taxa recoded using 9 minmax chi-squared bins**. Bayesian posterior probabilities are shown to the right of the nodes; posterior probabilities equal to 1.0 are indicated by black circles. Click here for file

Additional file 15 **Maximum likelihood tree calculated with the MULTIGAMMA model based on 2,729 amino acid positions (ALISCORE edited) of 49 metazoan taxa recoded using 9 minmax chi-squared bins**. Bootstrap support values larger than 50% are shown to the right of the nodes; 100% bootstrap values are indicated by black circles. Click here for file

Additional file 16 **Bayesian inference reconstruction with the CAT model based on 2,729 amino acid positions (ALISCORE edited) of 49 metazoan taxa recoded using 6 minmax chi-squared bins**. Bayesian posterior probabilities are shown to the right of the nodes; posterior probabilities equal to 1.0 are indicated by black circles. Click here for file

Additional file 17 **Maximum likelihood tree calculated with the MULTIGAMMA model based on 2,729 amino acid positions (ALISCORE edited) of 49 metazoan taxa recoded using 6 minmax chi-squared bins**. Bootstrap support values larger than 50% are shown to the right of the nodes; 100% bootstrap values are indicated by black circles. Click here for file

Additional file 18 **Bayesian inference reconstruction with the CAT model based on 2,729 amino acid positions (ALISCORE edited) of 49 metazoan taxa recoded using Dayhoff groups**. Bayesian posterior probabilities are shown to the right of the nodes; posterior probabilities equal to 1.0 are indicated by black circles. Click here for file

Additional file 19 **Maximum likelihood tree calculated with the MULTIGAMMA model based on 2,729 amino acid positions (ALISCORE edited) of 49 metazoan taxa recoded using Dayhoff groups**. Bootstrap support values larger than 50% are shown to the right of the nodes; 100% bootstrap values are indicated by black circles. Click here for file

Additional file 20 **Bayesian inference reconstruction with the CAT-BP model based on 2,729 amino acid positions (ALISCORE edited) of 49 metazoan taxa**. Consensus tree of all 16 chains. Bayesian posterior probabilities are shown to the right of the nodes; posterior probabilities equal to 1.0 are indicated by black circles. Click here for file

Additional file 21 **Maximum likelihood tree calculated with the GTR model based on 10,118 nucleotides (ALISCORE edited) of 49 metazoan taxa**. 20% of the alignment positions were removed based on high sitewise rates. Bootstrap support values larger than 50% are shown to the right of the nodes; 100% bootstrap values are indicated by black circles. Click here for file

Additional file 22 **Maximum likelihood tree calculated with the MtZoa+F model based on 2,456 amino acid positions (ALISCORE edited) of 49 metazoan taxa 10% of the positions were removed based on high sitewise rates**. Bootstrap support values larger than 50% are shown to the right of the nodes; 100% bootstrap values are indicated by black circles. Click here for file

Additional file 23 **Primer pairs and corresponding annealing temperatures used for successful amplification of mitochondrial genome fragments of *Flustra foliacea***. Click here for file

Additional file 24 **Species, classification and accession numbers of mitochondrial genome sequences used in the phylogenetic analyses**. Click here for file

Additional file 25 **Run parameters of the PhyloBayes analyses**. Click here for file
