# Supplementary material for: The complete mitochondrial genome of Flustra foliacea (Ectoprocta, Cheilostomata) - compositional bias affects phylogenetic analyses of lophotrochozoan relationships
Source: BMC Genomics. 2011 Nov 23;12:572. doi: 10.1186/1471-2164-12-572 (PMC3285623; doi:10.1186/1471-2164-12-572)

Maximum likelihood tree calculated with the GTR model based on 12,648 nucleotide positions (direct nucleotide alignment; ALISCORE edited) of 49 metazoan taxa. Bootstrap support values larger than 50% are shown to the right of the nodes; 100% bootstrap values are indicated by black circles.

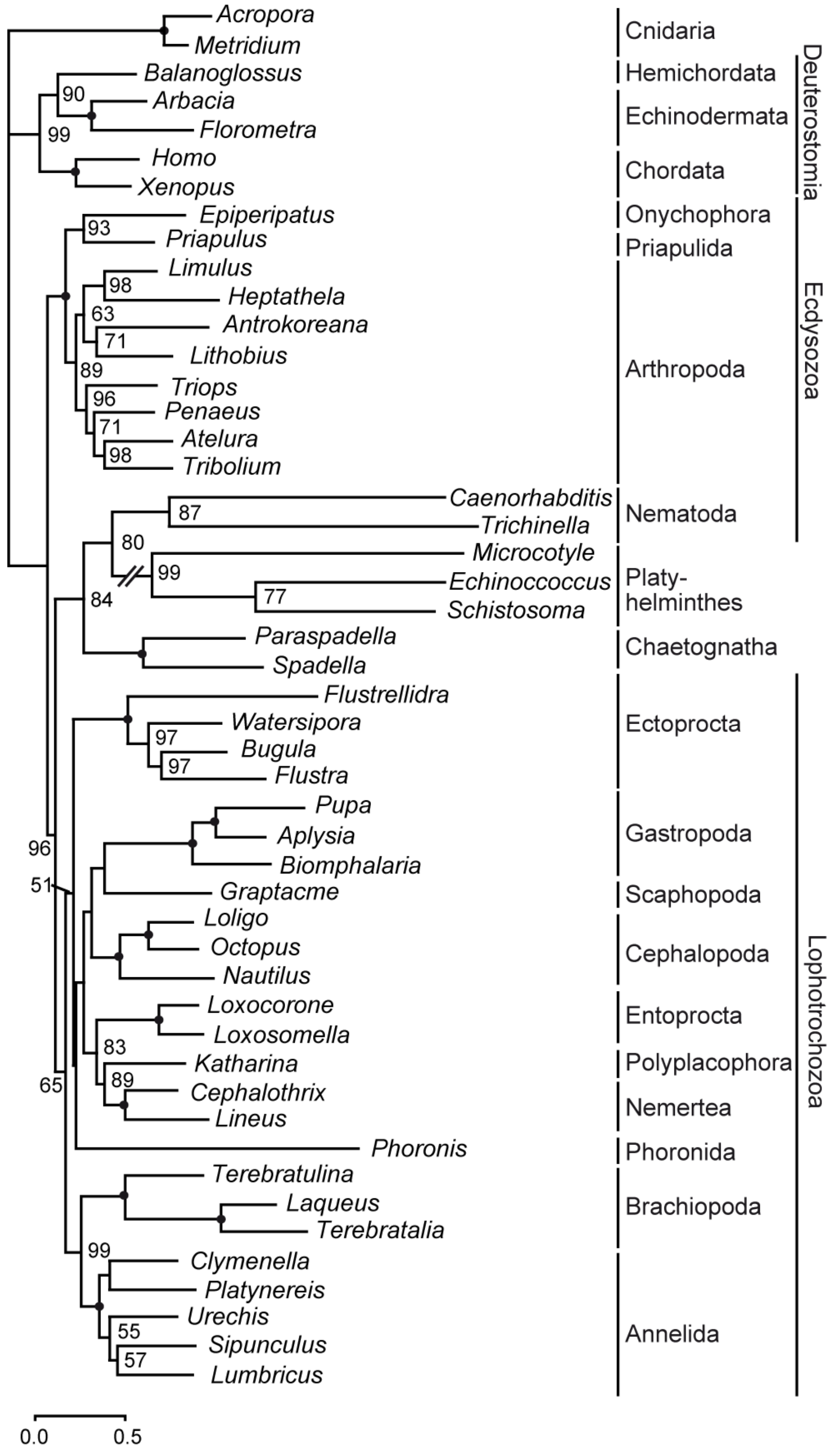

Supplement: Additional file 5 — Maximum likelihood tree calculated with the GTR model based on 12,648 nucleotide positions (direct nucleotide alignment; ALISCORE edited) of 49 metazoan taxa. Bootstrap support values larger than 50% are shown to the right of the nodes; 100% bootstrap values are indicated by black circles. [file 1471-2164-12-572-S5.PDF]
