# Supplementary material for: The complete mitochondrial genome of Flustra foliacea (Ectoprocta, Cheilostomata) - compositional bias affects phylogenetic analyses of lophotrochozoan relationships
Source: BMC Genomics. 2011 Nov 23;12:572. doi: 10.1186/1471-2164-12-572 (PMC3285623; doi:10.1186/1471-2164-12-572)

Maximum likelihood tree calculated with the nonstationary model implemented in nhPhyML-Discrete based on 10,629 nucleotide positions (ALISCORE edited) of 49 metazoan taxa. The maximum likelihood tree obtained with the nucleotide data set and the GTR model (Additional file 3) was used as starting tree.

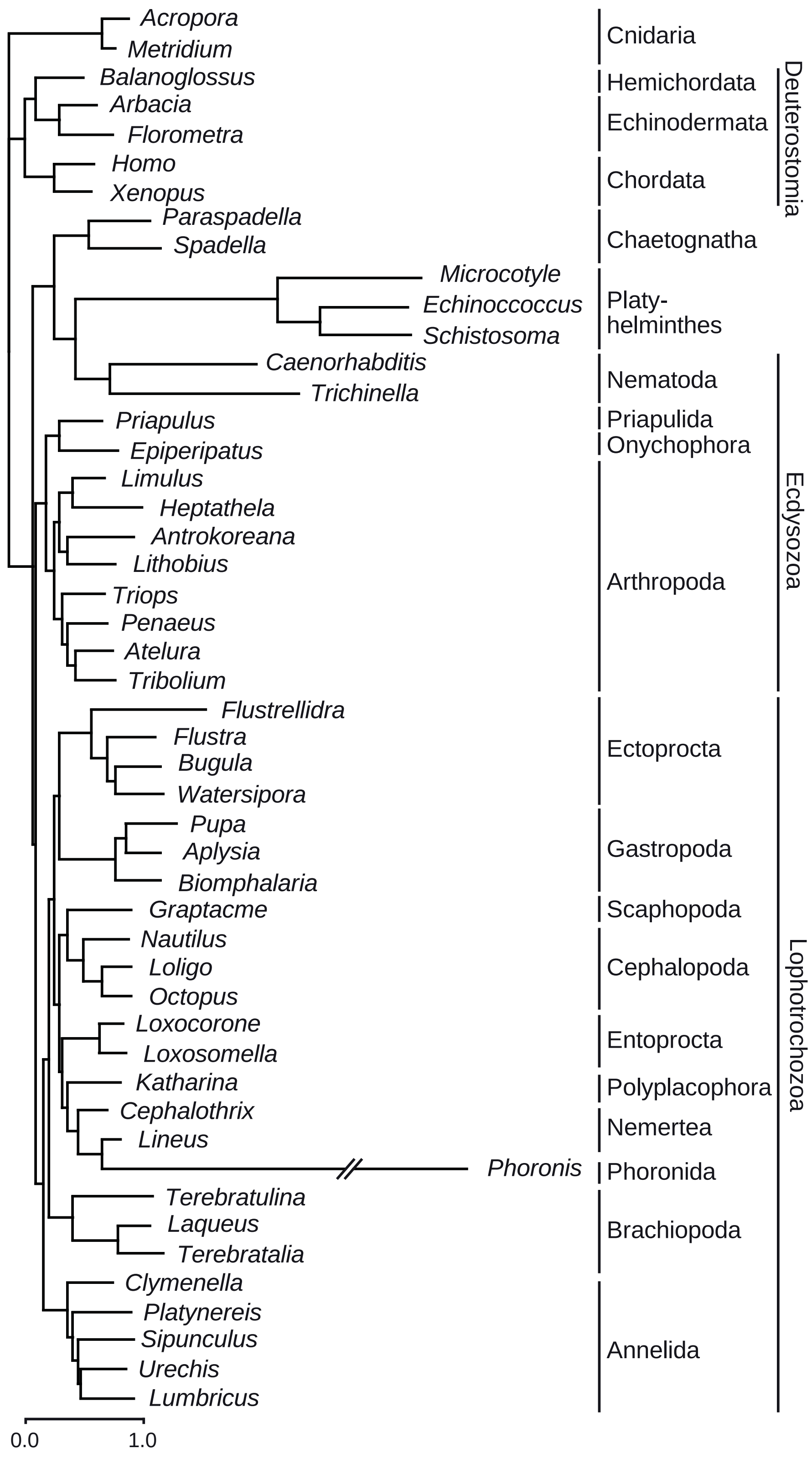

Supplement: Additional file 8 — Maximum likelihood tree calculated with the nonstationary model implemented in nhPhyML-Discrete based on 10,629 nucleotide positions (ALISCORE edited) of 49 metazoan taxa. The maximum likelihood tree obtained with the nucleotide data set and the GTR model (Additional file 3) was used as starting tree. [file 1471-2164-12-572-S8.PDF]
