# Supplementary material for: The complete mitochondrial genome of Flustra foliacea (Ectoprocta, Cheilostomata) - compositional bias affects phylogenetic analyses of lophotrochozoan relationships
Source: BMC Genomics. 2011 Nov 23;12:572. doi: 10.1186/1471-2164-12-572 (PMC3285623; doi:10.1186/1471-2164-12-572)

Maximum likelihood tree calculated with the nonstationary model implemented in nhPhyML-Discrete based on 10,629 nucleotide positions (ALISCORE edited) of 49 metazoan taxa. The Bayesian inference tree based on the amino acid sequences obtained with the CAT model (Fig. 5A) was used as starting tree.

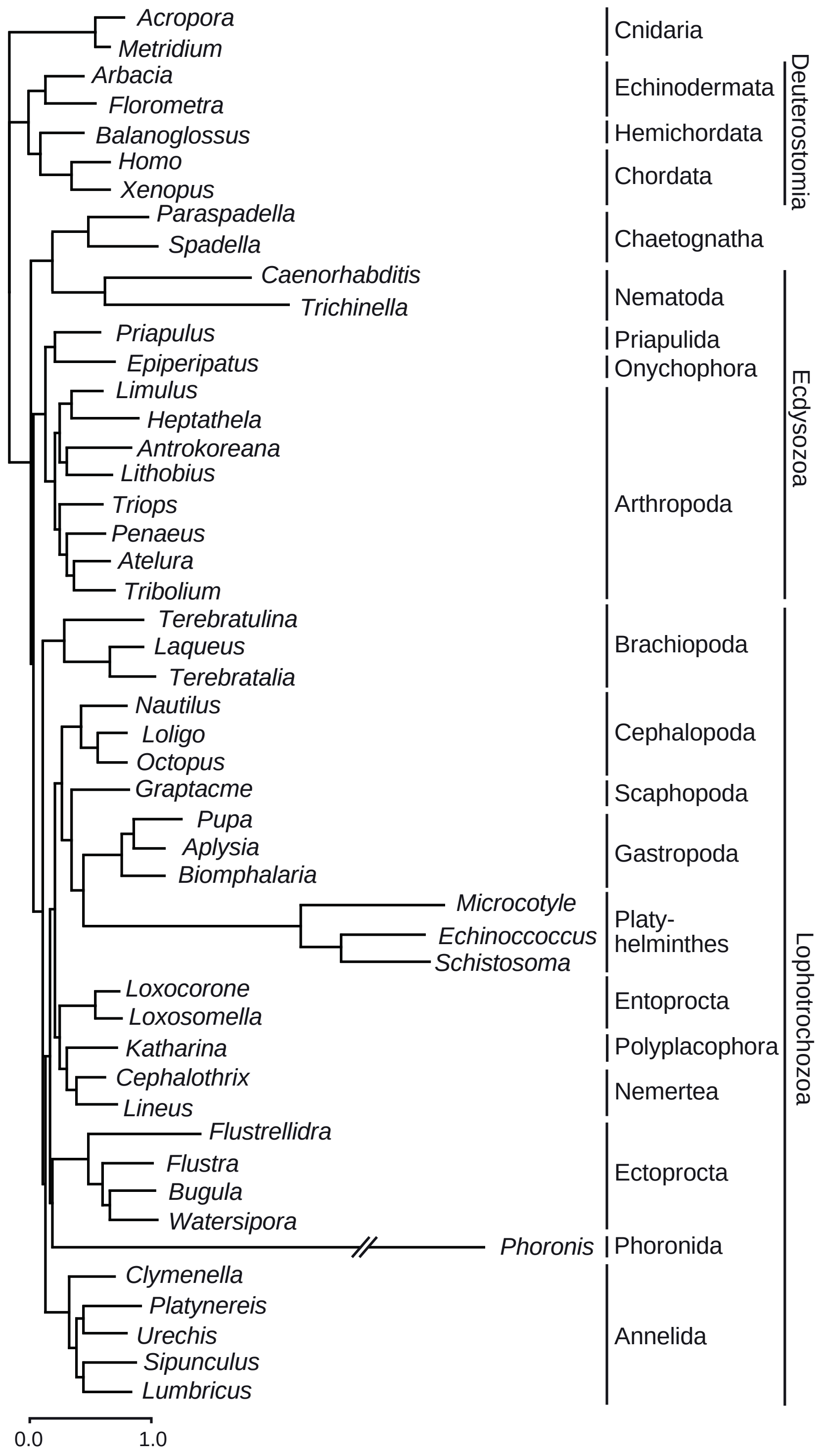

Supplement: Additional file 9 — Maximum likelihood tree calculated with the nonstationary model implemented in nhPhyML-Discrete based on 10,629 nucleotide positions (ALISCORE edited) of 49 metazoan taxa. The Bayesian inference tree based on the amino acid sequences obtained with the CAT model (Figure 5A) was used as starting tree. [file 1471-2164-12-572-S9.PDF]
