# Supplementary material for: The complete mitochondrial genome of Flustra foliacea (Ectoprocta, Cheilostomata) - compositional bias affects phylogenetic analyses of lophotrochozoan relationships
Source: BMC Genomics. 2011 Nov 23;12:572. doi: 10.1186/1471-2164-12-572 (PMC3285623; doi:10.1186/1471-2164-12-572)

Bayesian inference reconstruction with the CAT model based on 2,623 amino acid positions (ALISCORE edited) of 39 metazoan taxa (excluding the 10 taxa with the most significantly deviating amino acid composition). Bayesian posterior probabilities are shown to the right of the nodes; posterior probabilities equal to 1.0 are indicated by black circles.

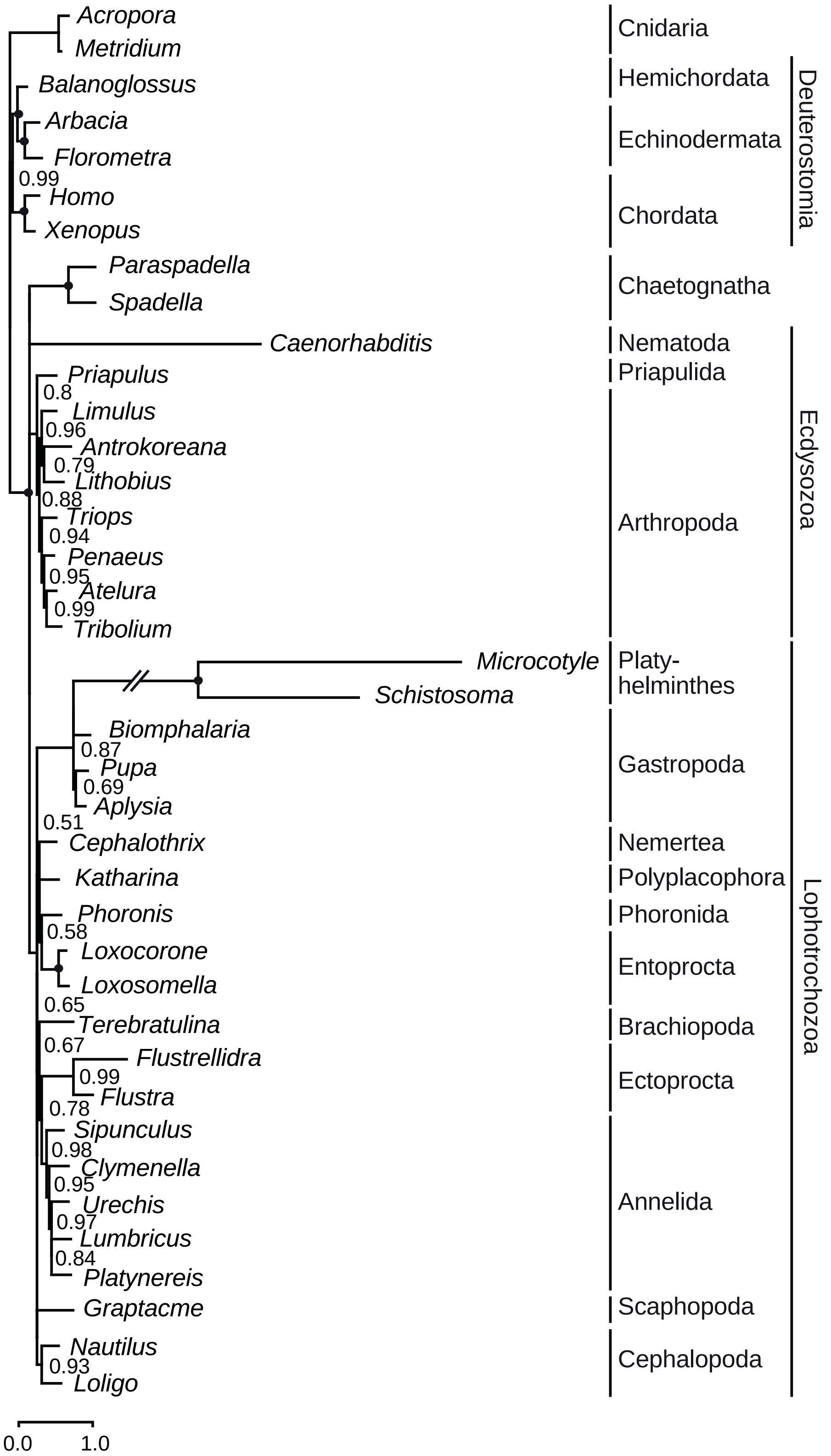

Supplement: Additional file 11 — Bayesian inference reconstruction with the CAT model based on 2,623 amino acid positions (ALISCORE edited) of 39 metazoan taxa (excluding the 10 taxa with the most significantly deviating amino acid composition). Bayesian posterior probabilities are shown to the right of the nodes; posterior probabilities equal to 1.0 are indicated by black circles. [file 1471-2164-12-572-S11.PDF]
