# Supplementary material for: The complete mitochondrial genome of Flustra foliacea (Ectoprocta, Cheilostomata) - compositional bias affects phylogenetic analyses of lophotrochozoan relationships
Source: BMC Genomics. 2011 Nov 23;12:572. doi: 10.1186/1471-2164-12-572 (PMC3285623; doi:10.1186/1471-2164-12-572)

Maximum likelihood tree calculated with the MtZoa+F model based on 2,623 amino acid positions (ALISCORE edited) of 39 metazoan taxa (excluding the ten taxa with the most significantly deviating amino acid composition). Bootstrap support values larger than 50% are shown to the right of the nodes; 100% bootstrap values are indicated by black circles.

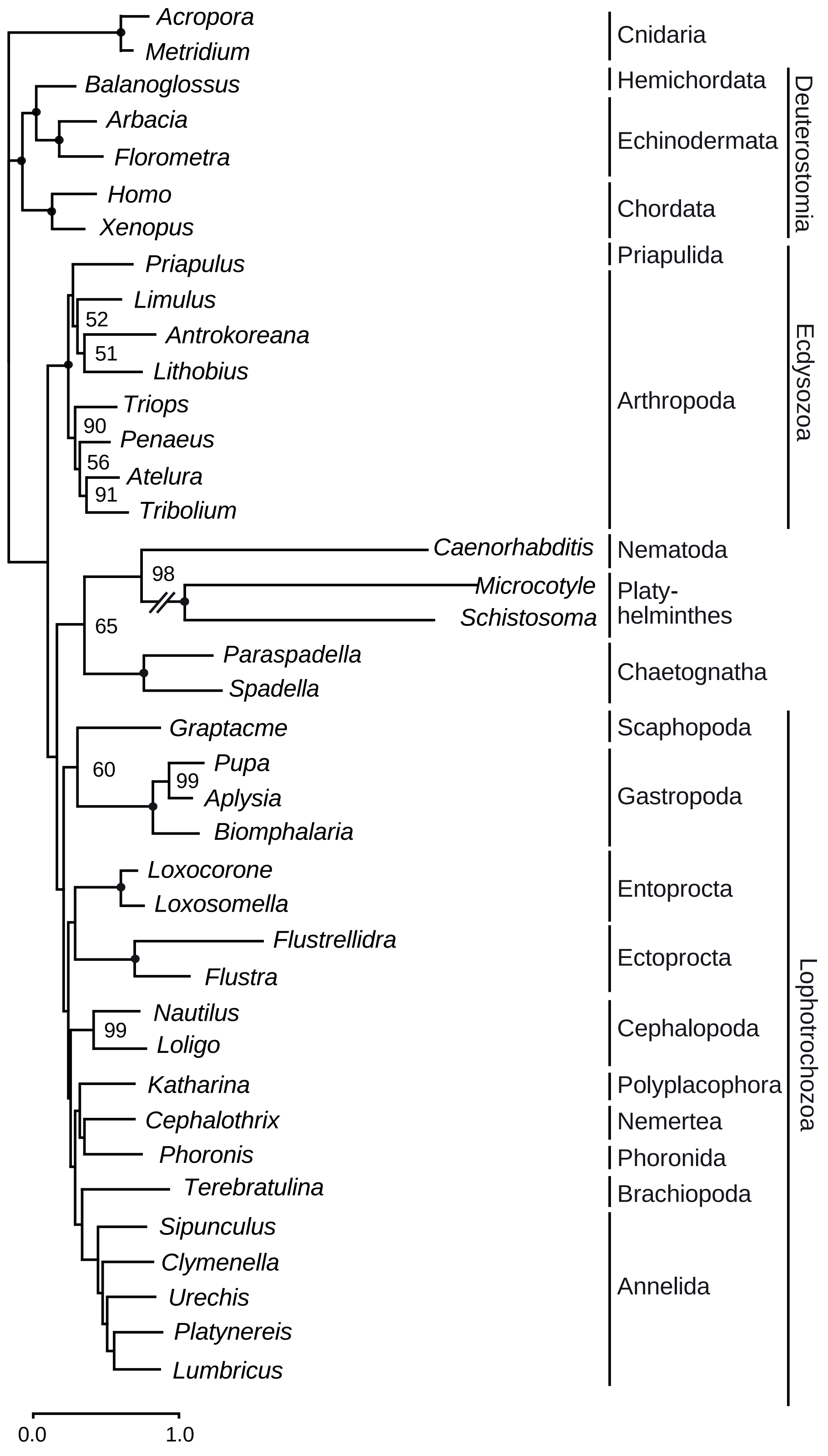

Supplement: Additional file 12 — Maximum likelihood tree calculated with the MtZoa+F model based on 2,623 amino acid positions (ALISCORE edited) of 39 metazoan taxa (excluding the ten taxa with the most significantly deviating amino acid composition). Bootstrap support values larger than 50% are shown to the right of the nodes; 100% bootstrap values are indicated by black circles. [file 1471-2164-12-572-S12.PDF]
