# Supplementary material for: The complete mitochondrial genome of Flustra foliacea (Ectoprocta, Cheilostomata) - compositional bias affects phylogenetic analyses of lophotrochozoan relationships
Source: BMC Genomics. 2011 Nov 23;12:572. doi: 10.1186/1471-2164-12-572 (PMC3285623; doi:10.1186/1471-2164-12-572)

Bayesian inference reconstruction with the CAT model based on 2,729 amino acid positions (ALISCORE edited) of 49 metazoan taxa recoded using Dayhoff groups. Bayesian posterior probabilities are shown to the right of the nodes; posterior probabilities equal to 1.0 are indicated by black circles.

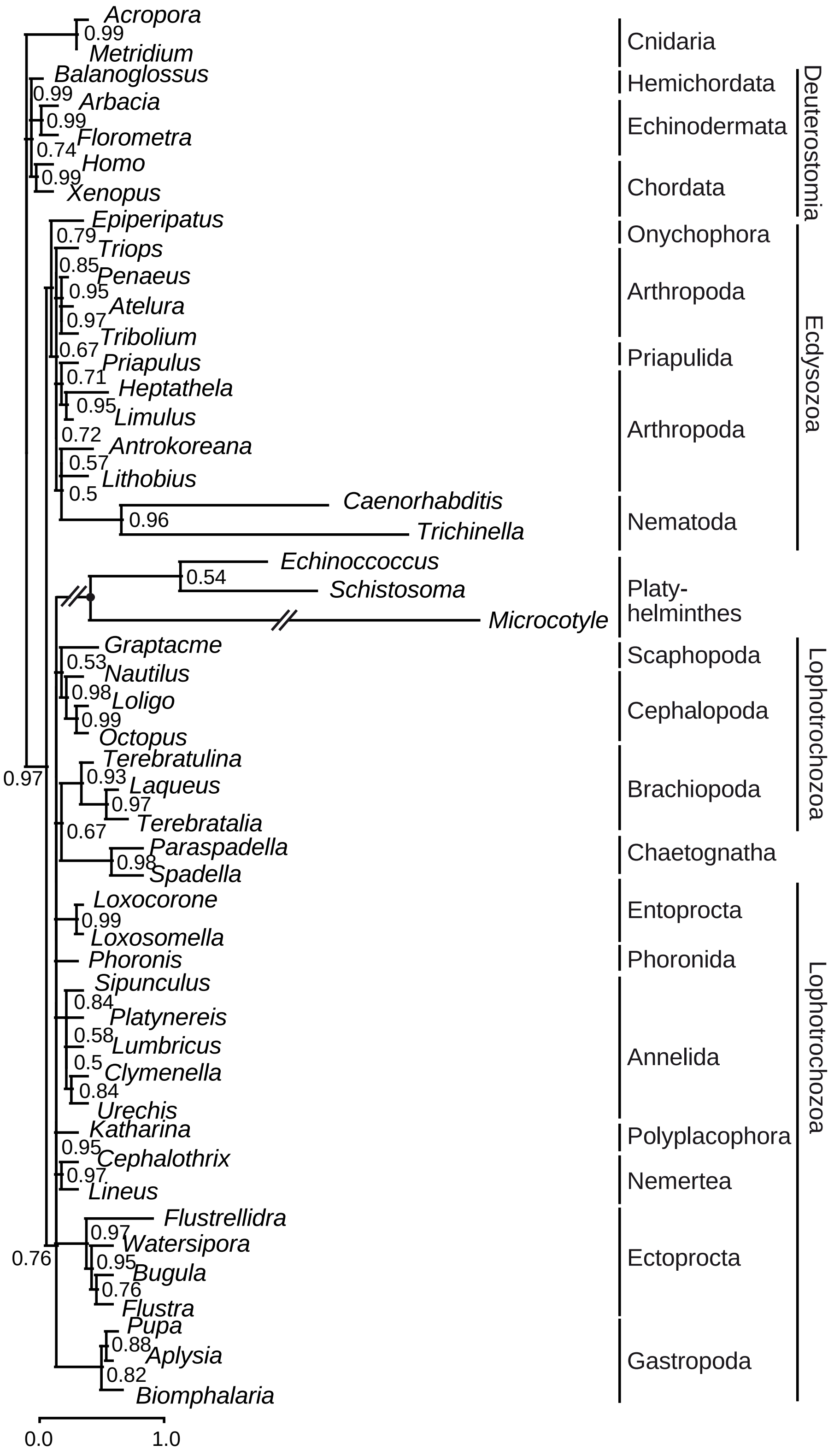

Supplement: Additional file 18 — Bayesian inference reconstruction with the CAT model based on 2,729 amino acid positions (ALISCORE edited) of 49 metazoan taxa recoded using Dayhoff groups. Bayesian posterior probabilities are shown to the right of the nodes; posterior probabilities equal to 1.0 are indicated by black circles. [file 1471-2164-12-572-S18.PDF]
